# Oncorhynchus mykiss *pax7* sequence variations with comparative analyses against other teleost species

**DOI:** 10.1186/s40064-015-1030-7

**Published:** 2015-06-17

**Authors:** Kalyan C Chapalamadugu, Brenda M Murdoch, Barrie D Robison, Rodney A Hill, Gordon K Murdoch

**Affiliations:** Department of Animal and Veterinary Science, University of Idaho, PO Box 442330, Moscow, ID 83844-2330 USA; Department of Biological Sciences, University of Idaho, 875 Perimeter MS 3051, PO Box 443051, Moscow, ID 83844-3051 USA; Ag Biotech 309, Department of Animal and Veterinary Science, University of Idaho, PO Box 442330, Moscow, ID 83844-3051 USA; University of South Florida, 12901 Bruce B. Downs, Tampa, FL 33612 USA

**Keywords:** Gene duplication, Paired box transcription factor-7, Rainbow trout, Satellite cells, Skeletal muscle

## Abstract

The paired box-7 (*pax7*) transcription factor expressed in satellite cells (SCs) is an essential regulator of skeletal muscle growth and regeneration in vertebrates including fish. Characterization of rainbow trout (Oncorhynchus mykiss) *pax7* gene/s may offer novel insights into skeletal myogenesis by SCs in this indeterminate growth species. Further, evaluation of promoters for *cis*-regulatory regions may shed light on the evolutionary fate of the duplicated genes. Employing standard PCR, cloning and computational approach, we identified and report complete coding sequences of two *pax7* paralogs of rainbow trout (rt); rt*pax7α* and rt*pax7β*. Both genes show significant identity in the nucleotide (97%) and the predicted amino acid (98%) sequences, and bear the characteristic paired domain (PD), octapeptide (OP) and homeodomain (HD) motifs. We further report several splice variants of each gene and nucleotide differences in coding sequence that predicts six putative amino acid changes between the two genes. Additionally, we noted a trinucleotide deletion in rt*pax7β* that results in putative serine elimination at the N-terminus and a single nucleotide polymorphism (SNP) in majority of the rt*pax7β* variants (6/10) that predicts an arginine substitution for a lysine. We also deciphered the genomic organization up to the first three exons and the upstream putative promoter regions of both genes. Comparative in silico analysis of both the trout *pax7* promoters with that of zebrafish *pax7* duplicates; zf*pax7a* and zf*pax7b;* predicts several important *cis*-elements/transcription factor binding sites (TFBS) in these teleost *pax7* promoter regions.

## Background

Growth and regeneration of skeletal muscle in vertebrates is mainly attributed to mitotically proficient adult muscle stem cells termed satellite cells (SCs) (Lepper et al. 2011; Moss and Leblond 1971; Motohashi and Asakura 2014; White et al. 2010). Activation of SCs by intrinsic cues such as hepatocyte growth factor (HGF) (Tatsumi et al. 1998) result in the generation of new myoblasts that terminally differentiate to myocytes and either fuse with the existing myofibers (hypertrophy) or among themselves to generate new myofibers (hyperplasia) (Collins et al. 2005; Mozdziak et al. 1997; Rowlerson et al. 2001). Alternately, the myogenic precursor cells can self-renew to replenish the intramuscular pool of SCs (Collins et al. 2005; Olguin et al. 2007). Depletion of *pax7* expressing SCs in skeletal muscle lead to compromised growth and skeletal muscle regeneration (Pascoal et al. 2013; Sambasivan et al. 2011).

While the myogenic program of SCs is primarily driven by myogenic regulatory factors (Megeney et al. 1996; Montarras et al. 2000; Smith et al. 1994), their maintenance, propagation and self-renewal in growing muscle have been attributed to the expression of *pax7* (Oustanina et al. 2004; Seale et al. 2004). The functional significance of *pax7* in the physiology of SCs is primarily understood through studies conducted in knock-out mice (Kuang et al. 2006; Oustanina et al. 2004; Relaix et al. 2006; Seale et al. 2004). Indeed, *pax7* is a widely accepted marker of SCs in vertebrates (Seale et al. 2000). Homozygous *pax7* null mice either suffer early postnatal lethality or grow to a small size, and show defective development of central nervous system and craniofacial muscles. Additionally, these mice suffer from defective postnatal skeletal muscle growth and regeneration due to a deficiency in the number of SCs (Kuang et al. 2006; Oustanina et al. 2004; Seale et al. 2000), suggesting that *pax7* deletion affects skeletal muscle development. Most recent studies using inducible knockout mouse models have further shown that expression of *pax7* is essential in mature skeletal muscle for effective regeneration and repair after injury (Günther et al. 2013; von Maltzahn et al. 2013). Together, *pax7* can be advanced as a key player in SCs biology with significant roles in skeletal muscle plasticity during both development and adult stages of higher vertebrates.

The role of SCs in fish skeletal muscle growth is well recognized (Koumans and Akster 1995; Marschallinger et al. 2009; Pascoal et al. 2013; Rossi and Messina 2014; Seger et al. 2011). Similar to mammals, SCs in various fish species express *pax7* (Devoto et al. 2006; Froehlich et al. 2013; Gotensparre et al. 2006; Marschallinger et al. 2009; Sibthorpe et al. 2006), and contribute to growth and regeneration of skeletal muscle (Seger et al. 2011) suggesting an evolutionarily important role of this transcription factor in vertebrate skeletal myogenesis. However, unlike mammals, teleost fish genomes contain more than one *pax7* gene. At least two *pax7* genes exist in zebrafish (Minchin and Hughes 2008), which has been attributed to the whole genome duplication early in the teleost lineage after divergence from their common mammalian ancestor (Jaillon et al. 2004). The salmonid genome may contain more copies of *pax7* (Gotensparre et al. 2006; Sibthorpe et al. 2006) due to another round of whole genome duplication around 88–103 Mya (Macqueen and Johnston 2014). Recent evidence from genomic sequencing studies in rainbow trout indicate that nearly half of the duplicated paralogs from this event are retained in the genome (Berthelot et al. 2014). Further evidence suggests that gene duplication in salmonids may also arise from localized gene duplication (Macqueen and Johnston 2006). Because of the importance of *pax7* in mediation of skeletal myogenesis by SCs, and its genetic complexity in teleost, an improved characterization of gene/s and promoter would add to a comprehensive understanding of the regulation and function of *pax7* in these species.

While growth of postnatal skeletal muscle in amniotes is primarily through hypertrophy, post-larval muscle accretion in salmonids is accomplished through both hyperplasia as well as hypertrophy (Mommsen 2001; Valente et al. 1998). Rainbow trout are an important global aquaculture species and an excellent animal model to study skeletal muscle growth that is mediated by SCs. However the structure and function of the *pax7* gene/s and the corresponding promoter/s is not well understood. In this study, we isolated multiple transcript variants of two rainbow trout *pax7* paralog genes (rt*pax7α* and rt*pax7β*), using skeletal muscle mRNA as the source of nucleotide sequences. Additionally, we deciphered the genomic organization of the first three exons and the associated 5′- flanking regions of both genes. Finally, an in silico analysis was performed to identify the potential *cis*-regulatory elements/TFBS in the putative promoter regions of each gene as compared to that of the zf*pax7a* and zf*pax7b* genes.

## Methods

### RNA isolation and RT-PCR

Skeletal muscle tissue from the hypaxial and epaxial regions of adult rainbow trout was collected following euthanization induced by 100 ppm of tricaine methanosulfonate (MS-222). Fish rearing, experimental sampling and handling procedures were approved by the University of Idaho Animal Care and Use Committee. All tissues were snap frozen in liquid N_2_ and stored at −80°C until RNA isolation. RNA was isolated using TRIzol reagent (Invitrogen, Carlsbad, CA, USA) following the manufacturer’s recommendations. Briefly, 1 ml of TRIzol was added to ~50 mg of ground muscle tissue and homogenized using a bead homogenizer at a frequency of 25 Hz for up to 1 min 30 s. RNA was separated by adding 0.2 ml of chloroform and centrifuged at 12,000×*g* for 10 min, at 4°C. The aqueous phase was collected and RNA was precipitated by adding 0.5 ml isopropyl alcohol and centrifuged at 12,000×*g* for 30 min at 4°C. RNA samples were finally washed twice in 1 ml of 75% ethanol and quantified on Nanodrop^®^ ND-1000 UV–Vis Spectrophotometer (Nanodrop Technologies, Wilmington, DE, USA) following manufacturer’s recommendations. Template quality was verified by visualization on a 1.5% formaldehyde agarose gel. A 2 µg of DNase I (Ambion, Foster City, CA, USA) treated RNA was then reverse transcribed using superscript III (Invitrogen) according to the manufacturer’s instructions, and the resultant cDNA served as a template for subsequent PCR amplification of *pax7* cDNA sequences.

### Isolation of *pax7* cDNA clones

Multiple nucleotide comparisons of the Atlantic salmon (Salmo salar) and Arctic char (Salvelinus alpinus) *pax7* cDNA variants (NCBI database) showed ~100% identity in the first exon and downstream of the initiator codon. Further, a rainbow trout *pax7*-like 3′-end enriched EST sequence (*CB493668*) also showed significant identity (94–98%) to various *pax7* variants of the above two species. Using the above information, a set of gene specific primers (GP7F, GP7R) was designed to amplify the putative complete protein coding sequences of trout *pax7* (Table 1). A touch down PCR was performed using Platinum® *Taq* DNA Polymerase (Invitrogen) on the cDNA prepared from total RNA of skeletal muscle as a template. Thermal cycler parameters comprised of an initial denaturation at 94°C for 3 min. Next six cycles each had a denaturation step at 94°C for 15 s, annealing step for 30 s where temperature was dropped by 1°C/cycle from 60 to 55°C and extension at 72°C for 2 min. Subsequent amplification was conducted for 29 cycles that comprised denaturation at 94°C for 15 s, annealing at 54°C for 30 s and extension at 72°C for 2 min. The reactions were completed with final extension performed at 72°C for 10 min. The PCR products were resolved on a 1% agarose gel and the appropriate amplicon was eluted from the gel using PureLink Quick gel extraction kit (Invitrogen) following manufacturer’s recommendations. Eluted PCR product was then subcloned into the pGEM-T Easy vector (Promega, Madison, WI) and transformed into One Shot TOP10 chemically competent *E. coli* (Invitrogen). Plasmid DNA was extracted using a GenElute™ HP Plasmid Miniprep Kit (Sigma-Aldrich, St. Louis, MO, USA) following manufacturer’s instructions and sequenced using an ABI 3730 capillary sequencer as per the manufacturer recommendations (Applied Biosystems, Foster City, CA, USA).

**Table 1:** List of primers used in PCR amplification reactions

Primer	Sequence	Length (bp)
GP7F	5′-ATG GCT ACT TTA CCA GGA ACA GT-3′,	23
GP7R	5′-TCA GTA GGC CTG TCC CGT CTC-3′	21
GP7a1	5′-GAA TGC CAT CGA TGC TAT GCT TTG TTT-3′	27
GP7a2	5′-CAC TAT CGT CGT CGT CAT CTT TCT TGC-3′	27
GP7a3	5′-TCT TAG CAA CAA TGT CAC CAT TGG TTT GG-3′	29
GP7a4	5′-GCA ACA ATG TCA CCA TTG GTT TGG TAA CT-3′	29
GP7b1	5′-GCT AAA GGG GTC TTC TTT TAC CCC ACA AA-3′	29
GP7b2	5′-TTT TGA GAG GAG ACA TTT CGT CAC ATC CT-3′	29
GP7b3	5′-AGA CCT AAG CAA ATG CGC GGA AAA ATA C-3′	28
GP7b4	5′-CTA TTT ATG CGA ATC GGT CCC ACA GTC T-3′	28
GP7b5	5′-GGG GGT TGA TAC TGT TCC ACA ATA AAC ATA-3′	30
GP7b6	5′-GGC GCA TCA TTC GAG GCA CTG TTC CTG GTA-3′	30
GP5R1	5′-TGG GCC ATC TCT ACT ATC TTG TGT CTG A-3′	28
GP5R2	5′-GGG GGT TGA TAC TGT TCC ACA ATA AAC ATA-3′	30

### 5′-rapid amplification of cDNA end to determine transcription start site (5′-RACE)

To identify the transcription start site (TSS), a rapid amplification of cDNA ends procedure was performed by using FirstChoice^®^ RLM-RACE Kit (Ambion). Following manufacturer’s instructions, decapped RNA from adult rainbow trout skeletal muscle was ligated to the manufacturer supplied oligonucleotide adaptor and reverse transcribed by employing either random hexamers or oligo(dT) primers. Subsequently, a touch-down nested PCR amplification was performed using reverse gene specific primers, GP5R1 (outer) and GP5R2 (inner) that were designed to anneal to a region around the start of PD region. Manufacturer supplied 5′-RACE adaptor primers served as forward primers. Thermal cycler parameters were as follows for the outer PCR; initial denaturation at 94°C–3 min. The next eight cycles each had a denaturation step at 94°C–30 s, annealing step for 1 min where temperature was dropped by 2°C for every two cycles from 64 to 61°C and extension at 72°C–1 min. The last 27 cycles had a denaturation step at 94°C–30 s, annealing step at 60°C–1 min and extension at 72°C–1 min. The reactions were completed with final extension performed at 72°C for 7 min. Subsequently performed inner PCR parameters were as follows; initial denaturation at 94°C–3 min. The next four cycles each had a denaturation step at 94°C–30 s, annealing step for 1 min where the temperature was dropped by 1°C for every two cycles from 66 to 65°C and extension at 72°C–1 min. The last 31 cycles had a denaturation step at 94°C–30 s, annealing step at 64°C–1 min and extension at 72°C–1 min. Final extension was performed for 7 min at 72°C.

### Isolation of genomic DNA and identification of *pax7* gene and promoter sequences

DNA was isolated from the skeletal muscle of adult rainbow trout. Briefly, ~100 mg of pulverized tissue was incubated at 37°C for 3 h in ten volumes of lysis buffer (10 mM Tris–Cl, pH 8.0, 0.1 M EDTA, pH 8.0, 0.5% SDS and 20 µg/ml DNase-free RNase A). Finally, proteinase K at a final concentration of 100 µg/ml was added to the lysate and incubated at 55°C overnight. DNA was then extracted by phenol–chloroform extractions followed by two washes in 75% ethanol. The quality of DNA was evaluated on a 1% agarose gel. Genome walker libraries were constructed using a GenomeWalker™ Universal kit (Clontech, Palo Alto, CA, USA) following the manufacturer’s instructions. Concisely, the genomic DNA was digested with blunt end restriction enzymes; EcoR V, *Dra* I, Pvu II or Stu I. The resultant DNA fragments were ligated to the Genome Walker adaptor supplied by the manufacturer (Clontech). Using a set of gene specific primers (reverse primers; gp7a1R, gp7a2R, gp7a3R and gp7a4R) (Table 1) in combination with the manufacturer supplied adaptor primers, the rt*pax7α* genomic DNA (gDNA) from the start of the 4th exon and up to 980 bp upstream of the initiator codon was isolated. Similarly, we isolated rt*pax7β* gDNA of 2,624 bp upstream of the initiator codon (gp7b5R and gp7b6R) (Table 1), and up to around the first three exons (using forward primers; gp7b1F, gp7b2F, gp7b3F and gp7b4F) (Table 1). All PCR were performed using Advantage 2 Polymerase Mix (Clontech) and thermal cycler parameters for outer PCR included 5 cycles at 94°C–25 s, denaturation; 72°C for 3 min, annealing and extension; followed by 32 cycles at 94°C–25 s, 67°C–3 min. A final extension at 67°C was performed for 7 min. All PCR products were analyzed on a 1.5% agarose gel, subcloned and sequenced as reported above.

### Phylogenetic analysis

Phylogenetic analysis was performed using phylogeny.fr pipeline (http://www.phylogeny.fr) with default parameters using rainbow trout putative Pax7 sequences (rtPax7Α and rtPax7β) along with those previously reported for other species; Atlantic salmon Pax7α (CAF02090), Atlantic salmon Pax7β (CAH04385), zebrafish Pax7a (NP_571400.1), zebrafish Pax7b (NP_001139621), tilapia Pax7 (LOC100708659, XP_003454575), tilapia Pax7 (LOC100696153, XP_003459869), stickleback Pax7 (1/2) (ENSGACP00000017071), stickleback Pax7 (2/2) (ENSGACP00000002231), medaka Pax7 (2/2) (ENSORLP00000005345), human Pax7 (DQ322591.1) and mouse Pax7 (NP_035169.1). Briefly, multiple alignments of amino acid sequences were accomplished using MUSCLE alignment program. Alignment curation was performed by Gblocks and phylogenetic analysis was performed by PhyML using a default substitution model (Dereeper et al. 2008).

### In silico analysis

Sequence analyses were performed using Vector NTI 11.5 advance (Invitrogen) at default parameters. Contiguous sequence (contig) alignment of nucleotide sequences was performed using the Contig assembly feature in Vector NTI 11.5 advance (Invitrogen). Gene homology searches were performed using the blast resources of NCBI database. Multiple comparisons of nucleotide and deduced amino acid sequences were performed using clustalW set at default parameters. Exon/intron boundaries were delineated using Spidey that is available at NCBI public domain (http://www.ncbi.nlm.nih.gov/spidey/). Prosite was used to perform pattern searches in the deduced amino acid sequences (Hulo et al. 2006). Putative *cis*-regulatory elements/transcription factor binding sites (TFBs) in gene regulatory/promoter regions were predicted using MatInspector software (http://www.genomatix.de), set at a matrix similarity threshold of 0.75.

## Results

### Isolation and characterization of two trout *pax7* paralogs

**Figure 1:**
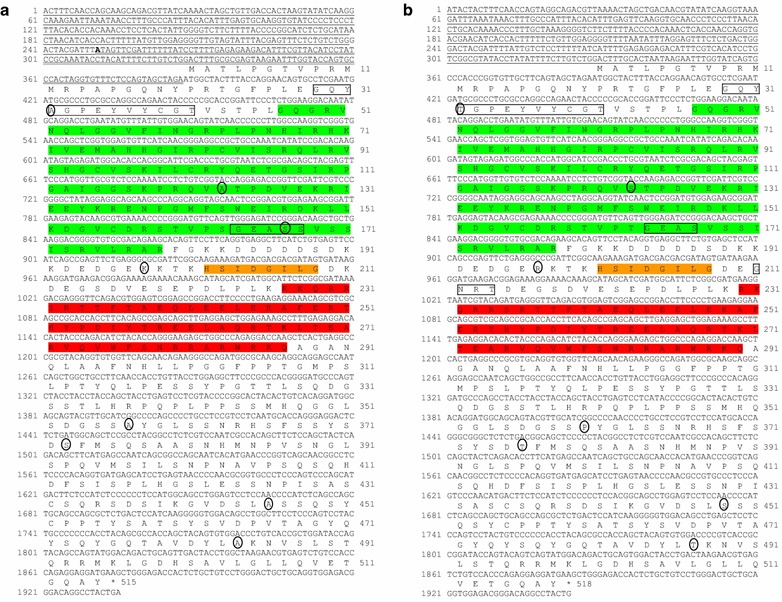
Nucleotide and deduced amino acid sequences of rainbow trout (rt) *pax7* cDNA forms: Sequences represent the longest transcripts cloned for each form, rt*pax7α* (**a**) and rt*pax7β* (**b**). Nucleotide sequences are numbered on the *left* and amino acid sequences are numbered on the *right*. Nucleotide sequence of 5′-UTRs is* underlined*. Shorter 5′-UTR of rt*pax7α* is *double underlined*. Conserved features of Pax7; paired domain (*green*), octapeptide (*orange*) and homeodomain (*red*) are highlighted. Amino acid residues that are alternately skipped are *boxed*. Amino acid residues that differ between the two forms are enclosed in *circle*.

Performing RT-PCR on cDNA synthesized from skeletal muscle total RNA produced an expected band of ~1,500 bp that was subcloned and sequenced. Subsequent analysis of all cloned cDNA yielded two distinct cDNA forms. Blast analysis showed high homology to *pax7* paralogs of other teleost fish; Atlantic salmon, Arctic char and zebrafish. We refer to these two transcript forms as rt*pax7α* and rt*pax7β*. A 5′-RACE protocol revealed two 5′-untranslated region (UTR) sequences for rt*pax7α* (identical sequence of variable length, 135 and 387 bp) and one for rt*pax7β* (388 bp). Assimilation of the respective 5′-UTR sequences with that of the longest transcript variant of rt*pax7α1* (Figure 1a) and rt*pax7β1* (Figure 1b) (Contig assembly application of vector NTI advance 11.5) resulted in 1935 and 1945 nucleotide long sequence, respectively. Conceptual translation of rt*pax7α1* (ATG at 388 nt.) and rt*pax7β1* (ATG at 389 nt.) resulted in putative proteins of 515 and 518 amino acids, respectively. Analyses of pattern identification using Prosite revealed the presence of conserved features in both putative protein forms that are characteristic of Pax7 protein; paired domain (PD), homeodomain (HD) and an octapeptide (OP) (Figure 1). A pairwise comparison of the nucleotide sequences showed that both forms share 83% identity in the 5′-UTR sequences, while the protein coding sequences share 97% identity. However, putative amino acid sequences are 98% identical. Therefore not all nucleotide polymorphisms in the coding regions resulted in amino acid substitutions. Indeed 47 polymorphic nucleotides were observed in the coding sequence, while only seven putative amino acid variations including a serine 167 deletion in rtPax7β1 (as compared to rtPax7α1) were observed between the two.

### Multiple splice variants

Multiple transcripts of each *pax7* gene differed in length due to an alternate indel of three regions, which suggests these transcripts represent alternate splice variants of each gene. Specifically, rt*pax7α* variants differed due to an indel of 39 bp (GQY(T)GPEYVYCGT), 15 bp (GEASS) or 12 bp (GNRT). Similarly, variants of rt*pax7β* differed by an indel of 39 bp (GQY(A)GPEYVYCGT), 12 bp (GEAS) or 12 bp (GNRT). Additionally, the majority of the rt*pax7β* clones sequenced (*6/10*) had a putative arginine substituted for lysine 197 due to an AAG to AGG transition, which suggests a potential allelic variation in the gene. The sequences of all the variants were deposited in the GenBank (Table 2).

**Table 2:** Splice variants of *pax7 *paralogs

cDNA variants	13 aa (±)ǂ	5/4 aa (±)ǂ	4 aa (±)ǂ	SNP	CDs (bp)*	Protein (aa)	Accession number
rt*pax7α*	GQYAGPEYVYCGT	GEASS	GNRT	K/R			
1	+	+	−	K	1,548	515	JQ303311
2	+	−	+	K	1,545	514	JQ303312
3	−	+	+	K	1,521	506	JQ303313
4	−	+	−	K	1,509	502	JQ303314

rt*pax7β*	GQYTGPEYVYCGT	GEAS-	GNRT	K/R	CDs (bp)*	Protein (aa)	Accession number
1	+	+	+	R/K	1,557	518	JQ303315
2	+	+	−	R	1,545	514	JQ303316
3	+	−	+	R/K	1,545	514	JQ303317
4	−	+	+	R/K	1,518	505	JQ303318
5	−	+	−	R	1,506	501	JQ303319
6	−	−	−	R	1,494	497	JQ303320
7	−	−	+	K	1,506	501	JQ303321

* Including stop, TGA; ^ǂ^indels corresponding to 13 aa, 5/4 aa and 4 aa, residues; *SNP* single nucleotide polymorphism in rtpax7β.

### Partial genomic characterization

**Figure 2:**
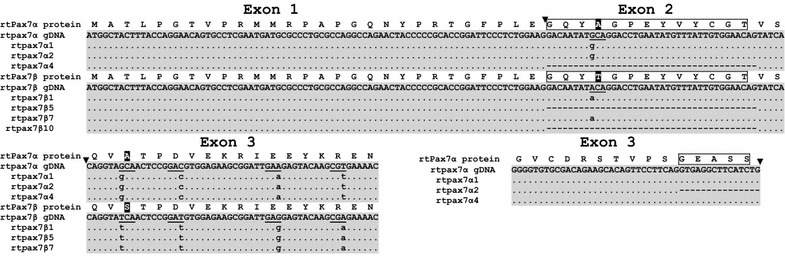
Partial genomic characterization of rainbow trout (rt) *pax7* cDNA forms; rt*pax7α* and r*tpax7β*: Multiple cDNA of both rt*pax7α* and rt*pax7β* are aligned with the respective genomic DNA (gDNA) sequences. Deduced amino acid sequences are shown on the *top* of each alignment. Exon boundaries are indicated by an *arrow* (▼). Polymorphic codons are *underlined*, and amino acid variations are highlighted in *black*. Amino acid residues corresponding to the insertion or deletion (indel) of the 39 bp in exon 2 or 15 bp in exon 3 are *boxed*. Identical nucleotide residues in all sequences are indicated by dotted line.

Employing a genome walker universal kit (Clontech) and a set of gene specific primers, genomic sequences corresponding to the 5′-UTR sequences and up to around the third exon were amplified (Figure 2). Sequenced partial gDNA of both rt*pax7α* and rt*pax7β* were individually aligned with corresponding transcripts using Spidey (NCBI) that delineated the exon/intron boundaries. While the substitution of threonine in rt*pax7β* for alanine 32 (rt*pax7α*) that occurs due to an ACA to GCA transition resides at the 5′-end of second exon, the substitution of serine in rt*pax7β* for alanine 123 (rt*pax7 α*), as a result of a GCA to TCA transversion, resides at the 3′ end of third exon (Figure 2). Although the presence of the trinucleotide sequence coding to serine 167 in rt*pax7α* was confirmed by our study, we did not sequence the gDNA corresponding to deleted serine 167 in rt*pax7β*. However, evidence from studies in other salmonids indicates the genomic fidelity of this deletion, given that it has been mapped to the gDNA of one of the two putative *pax7* paralogs reported for Atlantic salmon and Arctic char (Gotensparre et al. 2006; Sibthorpe et al. 2006). Further, the 39 bp indel maps to the 5′-end of second exon in both forms, while the 15 bp indel of rt*pax7α* maps to the 3′ end of third exon. In both cases, the splice junctions are consistent with the GT-AG rule. Further, the first introns of rt*pax7α* and rt*pax7β* were 1,309- and 1,333-bp, respectively, and had 80% sequence identity overall (data not shown). Similarly, the second introns were 550 and 617 bp long for rt*pax7α* and rt*pax7β*, respectively, and had 77% overall identity (data not shown). The 3rd intron sequenced only for rt*pax7α* was 216 bp. Although the genomic fidelity of 12 bp (GNRT) region is not verified in this study, previous studies indicate that this 12 bp indel maps to the 5′-end of fifth exon in salmonid *pax7*. These observations collectively indicate that rt*pax7α* and rt*pax7β* cDNAs are transcribed from two *pax7* genes that likely arose as a result of whole genome duplication.

### Phylogenetic associations

**Figure 3:**
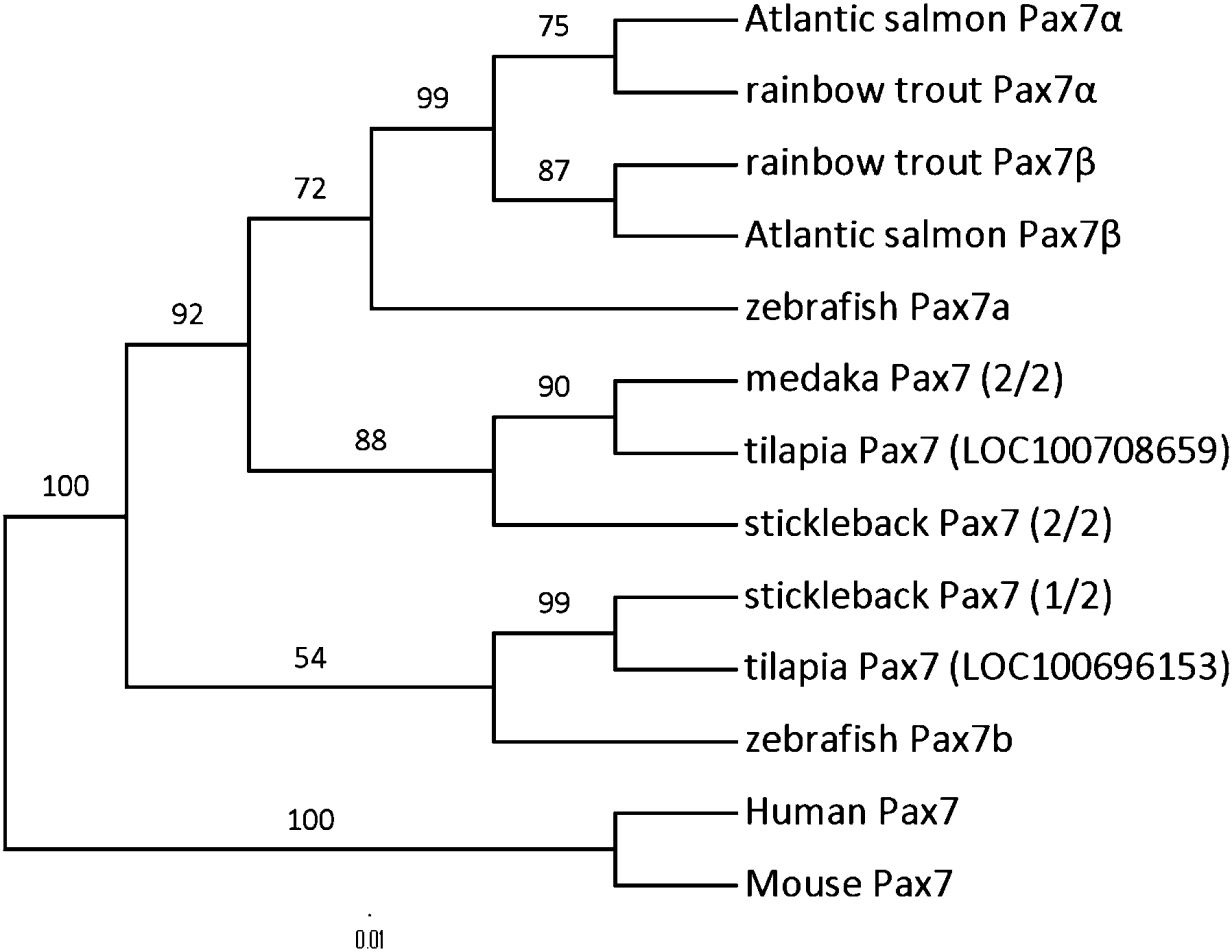
Phylogenetic analysis of Pax7 sequences of rainbow trout and other vertebrate species: Phylogenetic analysis of various vertebrate pax7 sequences including rainbow trout (Pax7α and pax7β) was performed using phylogeny.fr pipeline with default parameters. Amino acid sequences of rainbow trout pax7; pax7α and pax7β, Atlantic salmon pax7α (CAF02090), Atlantic salmon pax7β (CAH04385), zebrafish pax7a (NP_571400.1), zebrafish pax7b (NP_001139621), tilapia pax7 (LOC100708659, XP_003454575), tilapia pax7 (LOC100696153, XP_003459869), stickleback pax7 (1/2) (ENSGACP00000017071), stickleback pax7 (2/2) (ENSGACP00000002231), medaka pax7 (2/2) (ENSORLP00000005345), human PAX7 (DQ322591.1) and mouse PAX7 (NP_035169.1) were used. Branch support values (%) were reported on each branch.

All teleost *pax7* clustered differently from their mammalian orthologs and into two separate groups with high confidence (100%) (Figure 3). As suggested previously (Minchin and Hughes 2008), the observed topology indicates the existence of two *pax7* gene clades in teleost fish. The trout and Atlantic salmon *pax7* clustered into the same clade, and isoforms of both genes showed greater identity between the species than within the species isoform comparisons. This suggests a salmonid *pax7* gene paralogy that likely arose during a second round of presumed fish specific whole genome duplication. Clustering of zf*pax7a*, but not zf*pax7b*, sequences into same clade further suggests that rt*pax7α* and rt*pax7β* are co-orthologs of zebrafish *pax7a*.

### In silico analysis of *pax7* promoter regions

**Figure 4:**
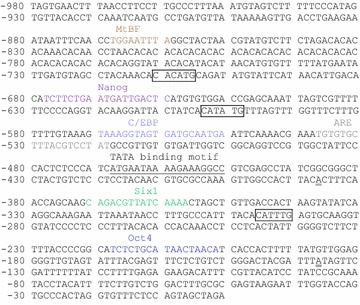
Nucleotide sequence of rainbow trout *pax7α* (rt*pax7α*) promoter region: Putative transcription factor binding sites are highlighted in *colors* and labeled above the consensus sequence. Putative E-box consensus sequences (CAN(T/A)TG) are *boxed*. TATA box sequence is *underlined*. Nucleotide positions are relative to initiator ATG (+1). Transcription start sites identified in this study are* double underlined*.

**Figure 5:**
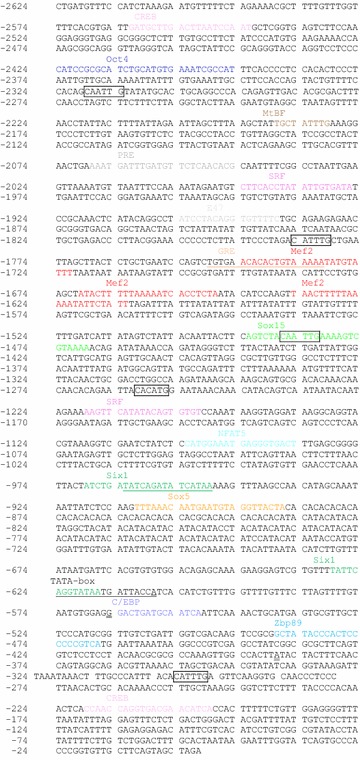
Nucleotide sequence of rainbow trout *pax7β* (rt*pax7β*) promoter region: Putative transcription factor binding sites elements are highlighted in *colors* and labeled above the consensus sequence. Putative E-box consensus sequences (CAN(T/A)TG) are boxed. TATA box sequence is *underlined*. Nucleotide positions are relative to initiator, ATG (+1). Transcription start site is* double underlined*.

Analysis of the rt*pax7α* (Figure 4) and the rt*pax7β* (Figure 5) promoter regions using MatInspector software showed consensus binding sites for several TFs in both the promoters. In the rt*pax7α* promoter region, a consensus TATA binding sequence is located between −452 and −468, 63 bases upstream of the TSS identified in this study. Important binding sites includes consensus sites for the following TFs: Octamer binding transcription factor-4 (Oct4), Nanog, androgen response element (ARE), muscle specific mitogen binding factor (MtBF), Sine-occulis homeobox 1 homolog (Six1) and CCAAT/enhancer binding protein (C/ebp) (Figure 4). In the rt*pax7β* promoter region, we found a consensus TATA binding sequence 219 bp upstream of the TSS identified in this study. However, a TATA-like sequence AATTAAATAA is also present 66 bp upstream of the TSS. Important binding sites includes consensus sites for the following TFs: a cAMP Responsive Element-Binding protein (Creb), Oct4, MtBF, E-box protein homodimer (E47), Myocyte specific enhancer factor-2 (Mef2), Six1, SRY related HMG box factors -5 (Sox5) and -15 (Sox15), glucocorticoid response element (GRE), progesterone response element (PRE), Nuclear factor of activated T-cells 5 (Nfat5), Krüppel-like zinc-finger transcription factor (Zbp89) and C/ebp (Figure 5). Also, both promoters have multiple putative E-boxes with consensus CAN(T/A)TG sequence.

**Figure 6:**
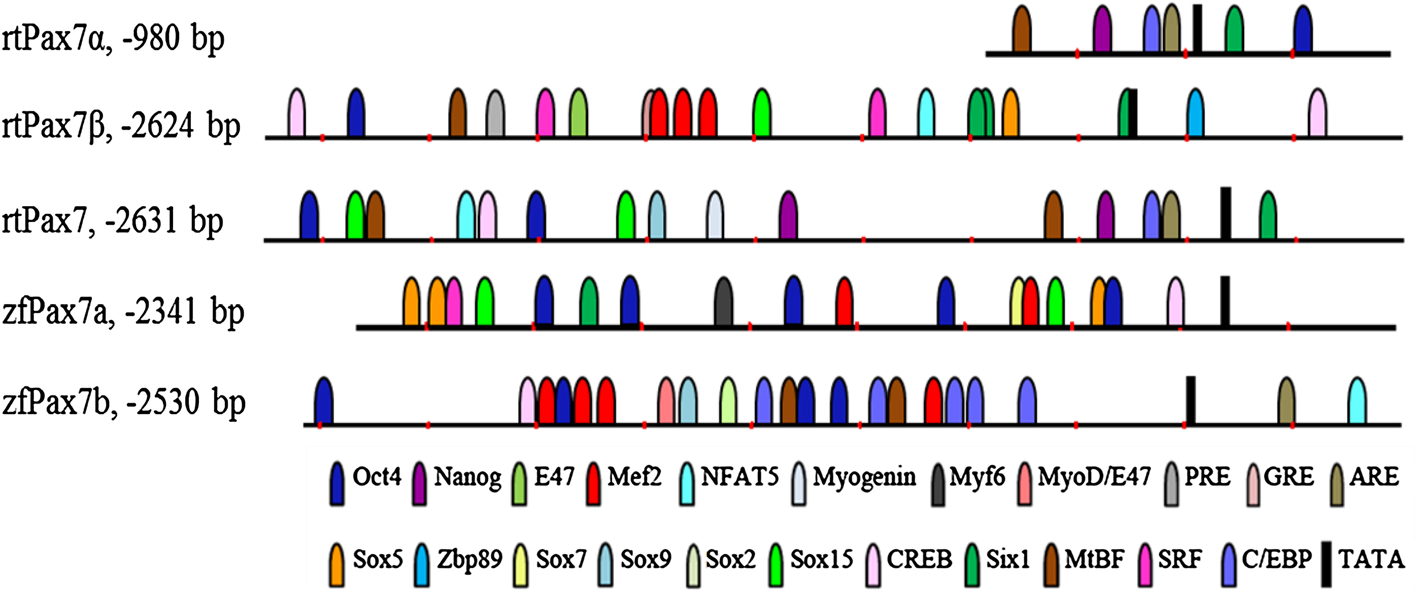
Comparative in silico analysis of fish *pax7* promoter sequences: Nucleotide sequences upstream of the initiator codon of rt*pax7α*, rt*pax7β*, trout *pax7* promoter, *rtpax7* (FJ713022.1), zebrafish *pax7*; *zfpax7a* (NC_007122.5 Reference Zv9 Primary Assembly) and *zfpax7b* (NC_007134.5 Reference Zv9 Primary Assembly) were analyzed for the presence of relevant transcription factor binding sites, and comparative promoter maps are presented. Each *cis*-element is represented by a *different color* as indicated in the key and every element is presented relative to its position within each promoter.

Comparative in silico analysis of trout *pax7* promoters with that of the putative zebrafish promoter regions; zf*pax7a* and zf*pax7b*, show that several if not all, TFBS are similarly present in these two zebrafish *pax7* promoter regions (Figure 6). Binding sites for Oct4 and Six1 (except zf*pax7b*) are observed in all promoters analyzed, although multiple Oct4 sites are present in zebrafish *pax7* promoters. All promoters with the exception of rt*pax7α* also have more than one Sox binding site. Specifically, Sox15 and Sox5 binding site/s are predicted in both rt*pax7β* and zf*pax7a* promoter regions. Additionally, binding sites for Sox2 and Sox9 transcription factors are predicted in the zf*pax7b* promoter region. Although the various promoters differ in the genetic nature of these *cis*-elements, the presence of Sox binding elements appears to be a common feature in these fish promoters as the rainbow trout *pax7* promoter region (sequence that is highly similar to rt*pax7α*) also have binding sites for Sox9 and Sox15. Comparative analyses further show that these fish promoters have MRF binding sites. Although we did not find putative sites for MRFs within the sequences we cloned, a myogenic factor 6 (Myf6) binding site in zf*pax7a* promoter and a MyoD/E47 heterodimer binding site in zf*pax7b* promoter were identified. Also, an ARE that is observed in rt*pax7α* is also present in zf*pax7b* promoter. However, a Zbp89 binding site is identified only in rt*pax7α*, while rt*pax7β* and zf*pax7b* promoters each have one Nfat5 binding site. Although the binding and functional relevance have to be experimentally derived, presence of these *cis*-regulatory elements in trout promoters suggests some degree of evolutionary conservation in *pax7* gene regulation.

## Discussion

Paired box-7 transcription factor has been implicated in vertebrate skeletal muscle growth and development. It is expressed in skeletal muscle SCs and plays a principal regulatory role in adult skeletal myogenesis. In this study, we identified two *pax7* genes (rt*pax7α* and rt*pax7β*) and their putative splice variants in rainbow trout. Further, we sequenced the promoter regions of both genes and performed an in silico analysis to identify the putative TFBS in the promoter regions of both genes as compared to duplicate zebrafish *pax7* gene promoters.

An important finding of this study was the identification and characterization of two highly homologous *pax7* genes in rainbow trout, suggesting both genes likely arose from a salmonid whole genome duplication event (Macqueen and Johnston 2014). With the recent availability of the trout genome sequences (Berthelot et al. 2014), one recent study reported the presence of three mammalian *pax7* co-orthologs in rainbow trout genome: *pax7a1*, *pax7a2* and *pax7b1* (Seiliez et al. 2014). Comparison of our sequences with the partial cDNA sequences annotated in this study showed nucleotide identities of 97.7% between rt*pax7α and pax7a2* and 99.7% between rt*pax7β* and *pax7a1*, indicating that we have successfully identified the two genes of the *pax7a* clade of rainbow trout. As also reported (Seiliez et al. 2014), our phylogenetic analysis further support this observation because both rt*pax7α* and rt*pax7β* cladistically belong with zf*pax7a*. Although our analysis also supports the existence of additional *pax7* gene/s in second clade, we did not identify sequences that are homologous to *pax7b1* in this study. This is however not surprising given the sequence dissimilarities between *pax7a* members and *pax7b1* at the extreme 5′ end of the coding sequence where we targeted our forward primer to amplify rt*pax7α* and rt*pax7β* sequences.

Both *pax7* paralogs also express multiple splice variants in the adult skeletal muscle, which adds another layer of complexity to *pax* gene function in trout. Expression of splice variants appears to be a common mode of *pax* gene regulation (Barber et al. 1999; Pritchard et al. 2003; Ziman and Kay 1998). For instance in mice, the single gene expresses 4 alternate spliced transcripts in adult skeletal muscle tissue (Ziman and Kay 1998). These variants show different expression levels and altered DNA binding and transactivation properties (Du et al. 2005; Ziman and Kay 1998). Similarly, expression of multiple *pax7* splice variants has been reported in zebrafish, Atlantic salmon and Arctic char (Gotensparre et al. 2006; Seo et al. 1998; Sibthorpe et al. 2006). Moreover, the alternately skipped 13 amino acid residues at the N-terminus appear to be a common feature of salmonid fish Pax7. Additionally, the substitution of threonine for alanine 32 and serine for alanine 123 in rtPax7α resulted in the inclusion of two additional casein kinase—II phosphorylation sites with a consensus sequence of S/T-X-X-D/E. CK2 is a common serine threonine kinase protein and phosphorylates multiple factors involved in vertebrate myogenesis (Johnson et al. 1996; Molkentin et al. 1996; Winter et al. 1997). Also, the deletion of GNRT residues from either form also results in the elimination of an Asn-glycosylation site that has a consensus, Asn-X-S/T-Y, sequence (NRTD). These features are particularly interesting as the previous studies on Pax3/7-FKHR fusion proteins revealed that a *cis*-acting functional transcriptional repression domain exists at the N-terminus of both Pax3 and Pax7 (Bennicelli et al. 1999). Although the relevance of these variations in Pax7 post-translational modifications, DNA binding affinity and transactivation properties has to be functionally determined; the production of multiple splice variants may provide enormous diversity in Pax7 target gene regulation.

Extant paralog genes may develop unique expression patterns and acquire diverse fates including gain of novel function (neo-functionalization) or retain a portion of the original gene function (sub-functionalization) (Conant and Wolfe 2008). These diverse fates of the paralogs can at least in part arise by divergence in their regulatory/promoter regions (Van Hellemont et al. 2007). Recently, Seiliez et al. (2014) showed that *pax7* paralogs of rainbow trout differ in their expression pattern during the course of satellite cell conversion to myocytes that has been attributed to the differential epigenetic histone modifications in the *pax7* gene loci. Our in silico examination reveals putative binding sites for several important TFs in the promoter regions of both trout *pax7* genes. Comparative analyses suggest that several of these sites are also present in zebrafish *pax7* gene promoters. The presence of one or more Oct4 binding sites in all promoters analyzed in the present study suggests an evolutionarily conserved role of this transcription factor in *pax7* gene regulation. Oct4 is one of the master inducers of pluripotency in embryonic stem cells, and studies show that Oct4 binds mouse *pax7* promoter and functions as a transcriptional repressor (Lang et al. 2009). Further, the presence of a putative binding site for Nanog that is also an inducer of pluripotency in close proximity to TSS of rt*pax7α* is especially interesting, as co-expression of Nanog with Oct4 significantly inhibits myogenic cell differentiation, although binding of Nanog itself to *pax7* promoter was not observed (Lang et al. 2009). Further, putative binding sites for various members of Sox family were observed in these fish *pax7* promoters. Past studies, although primarily in mammalian models, showed that satellite cells express various *sox* genes. Sox8 has been implicated in maintaining the satellite cell progenitor population (Schmidt et al. 2003). In vitro cell culture studies using P19 cell lines showed that both Sox15 and Sox7 influence Pax3/Pax7 expression and myogenesis (Savage et al. 2009). Also, genetic knock-out studies in mice show that Sox15 is essential for adult skeletal muscle regeneration (Lee et al. 2004). The identification of a putative binding site for Zbp89 that binds human *pax7* promoter was also noted in rt*pax7β* promoter (Salmon et al. 2009). Zbp89 is expressed in skeletal muscle of various mammalian species (Merchant et al. 1996) and acts as a *pax7* transcriptional repressor while enhancing myogenic differentiation (Salmon et al. 2009). While the presence of commonality in the genetic nature of *cis*-elements in these fish promoters suggests significant degree of conservation in *pax7* gene regulation at the promoter level, presence of unique *cis*-elements in each of trout *pax7* promoter regions may point to the potential differences in their regulation. Nevertheless, a detailed functional characterization is required to unambiguously ascertain the role of these and other TFBS in trout *pax7* promoters.

## Conclusions

In conclusion, the sequence information of rainbow trout putative *pax7* paralog genes and their corresponding splice variants will facilitate future studies designed to characterize the tissue and stage-specific expression profiles of these transcript variants and their consequent function. Although the physical presence of the reported TFBS in these teleost *pax7* promoters may not necessarily confer binding by the corresponding TFs, existing literature report the expression of many of these TFs in the context of vertebrate skeletal myogenesis that is mediated by SCs, which indicates a functional relevance of these TFs in teleost *pax7* gene regulation. Therefore, future studies that delineate the minimal promoter regions and experimental characterization of these putative *cis*-elements would shed more light on the functional aspects of *pax7* gene regulation.
